# Pancreatic Enzyme Replacement and Nutritional Support With *n﻿ab*-Paclitaxel-based First-Line Chemotherapy Regimens in Metastatic Pancreatic Cancer

**DOI:** 10.1093/oncolo/oyad101

**Published:** 2023-05-08

**Authors:** Guido Giordano, Raffaele Ivan Cincione, Francesca Losavio, Tiziano Senia, Arianna Aquilini Mummolo, Mario Pacilli, Vincenzo Lizzi, Giuseppina Bruno, Annamaria Piscazzi, Vincenza Conteduca, Matteo Landriscina

**Affiliations:** Unit of Medical Oncology and Biomolecular Therapy, Department of Medical and Surgical Sciences - Policlinico Riuniti, University of Foggia, Foggia, Italy; University Service of Diet Therapy and Metabolic Diseases, Department of Clinical and Exprerimental Medicine - Policlinico Riuniti, University of Foggia, 71122 Foggia, Italy; University Service of Diet Therapy and Metabolic Diseases, Department of Clinical and Exprerimental Medicine - Policlinico Riuniti, University of Foggia, 71122 Foggia, Italy; University Service of Diet Therapy and Metabolic Diseases, Department of Clinical and Exprerimental Medicine - Policlinico Riuniti, University of Foggia, 71122 Foggia, Italy; University Service of Diet Therapy and Metabolic Diseases, Department of Clinical and Exprerimental Medicine - Policlinico Riuniti, University of Foggia, 71122 Foggia, Italy; General Surgey Unit, Department of Medical and Surgical Sciences - Policlinico Riuniti, University of Foggia, 71122 Foggia, Italy; General Surgery Unit - Policlinico Riuniti, 71122 Foggia, Italy; Unit of Medical Oncology and Biomolecular Therapy, Department of Medical and Surgical Sciences - Policlinico Riuniti, University of Foggia, 71122 Foggia, Italy; Unit of Medical Oncology and Biomolecular Therapy, Department of Medical and Surgical Sciences - Policlinico Riuniti, University of Foggia, 71122 Foggia, Italy; Unit of Medical Oncology and Biomolecular Therapy, Department of Medical and Surgical Sciences - Policlinico Riuniti, University of Foggia, 71122 Foggia, Italy; Unit of Medical Oncology and Biomolecular Therapy, Department of Medical and Surgical Sciences - Policlinico Riuniti, University of Foggia, 71122 Foggia, Italy

**Keywords:** metastatic pancreatic cancer, PERT, nutritional support, *nab*-paclitaxel, first-line therapy

## Abstract

**Background:**

At diagnosis, more than 80% of patients with pancreatic cancer (PC) suffer from significant weight loss due to malnutrition which is a major concern for patient management, and this may negatively impact treatment outcomes and patient prognosis.

**Patients and Methods:**

We performed an observational, retrospective study on patients with metastatic PC (mPC) undergoing first-line chemotherapy with *nab*-Paclitaxel containing schedules and receiving or not receiving nutritional support (NS) and pancreatic enzyme replacement therapy (PERT) to investigate their relevance in this setting.

**Results:**

We observed that PERT and ancillary dietary interventions are related to longer overall survival (OS; median: 16.5 vs. 7.5 months, *P < .*001) and have a significant, independent, prognostic impact for better outcomes (*P = .*013), independently from the therapeutic regimen. Furthermore, PERT and NS prevented weight loss during chemotherapy and obtained an improvement of nutritional parameters such as phase angle and free-fat mass index, after 3 months of anticancer treatment. Consistently, the positive impact on OS correlated also with the prevention of Karnofsky performance status deterioration and a lower incidence of maldigestion-related symptoms.

**Conclusions:**

Our data suggest that an early and well-conducted NS in patients with mPC may impact on survival and preserve performance status, thus improving quality of life.

## Teaser

Most patients with pancreatic cancer suffer significant weight loss due to malnutrition, which negatively affects outcomes. This article describes results of a study of patients with metastatic pancreatic cancer undergoing first-line chemotherapy with *nab*-paclitaxel either receiving or not receiving nutritional support and pancreatic enzyme replacement therapy (PERT).

**Implications for Practice**

Combined intervention with nutritional support and pancreatic enzyme replacement therapy could positively impact on pancreatic cancer patients both in terms of performance status and clinical outcomes.

## Introduction

Pancreatic cancer (PC) is one of the malignancies with worst prognosis, being the 7th leading cause of cancer death in industrialized countries.^1^ The high mortality rate due to disease aggressiveness and difficulties in early diagnosis has been little improved by recent advancements in detection and management.^2,3^ So little that poor prognosis resulted in a similar number of deaths (466 003) and diagnosed cases (495 774) across 185 countries in 2020.^4^ Most of patients present locally advanced, unresectable, or metastatic disease at the time of diagnosis; therefore, treatment aims only to control symptoms, prolong survival, and improve quality of life.^2^

In this setting, chemotherapy represents the only therapeutic choice, but survival remains poor. In the last decade, the introduction of *nab*-paclitaxel (Nab-P) in combination with gemcitabine has prolonged the overall survival (OS) in metastatic PC (mPC) treatment.^5^ Recently, a more aggressive, four-drug regimen of cisplatin, Nab-P, gemcitabine, and capecitabine (PAXG) has showed promising results in this setting in a phase II study^6^ and has been recommended by the Italian Association of Medical Oncology guidelines as one of the first-line therapeutic options.^7^

At diagnosis, more than 80% of patients with PC suffer from significant weight loss due to malnutrition, induced by metabolic, psychological and gastrointestinal factors, including tumor cytokines-induced anorexia, elevated resting energy expenditure, gastric/biliary obstruction, malabsorption, and treatment side effects.^8^ In such a context, malnutrition is a major concern for patient management and may negatively affect treatment outcomes and patient prognosis. In fact, it is associated with longer hospitalization, increased risk for complications, lower response to treatment, poor quality of life, and increased morbidity and mortality.^9^ A frequent occurrence is pancreatic exocrine insufficiency due to the cancer location at the head of the pancreas. It leads to reduced secretion of pancreatic enzymes during meals, maldigestion, and secondary malnutrition and may be managed with oral pancreatic enzyme replacement therapy (PERT).^2,10^ Identifying patients at risk of malnutrition may favor prompt and adequate supportive interventions, improve oncologic outcomes, and patient well-being.^8^

We performed an observational, retrospective study on a cohort of 106 consecutive patients with mPC undergoing first-line chemotherapy with Nab-P containing schedules and receiving or not receiving nutritional support (NS) and PERT to investigate their relevance in this setting.

## Patients and Methods

### Study Overview and Population

This is an observational, retrospective, single-institution study including consecutive patients with mPC treated in daily clinical practice with Nab-P-based first-line chemotherapy between January 2016 and January 2022. Inclusion criteria were represented by (1) age ≥18 years, (2) cytological or histological diagnosis of PC, (3) stage IV disease, (4) first-line chemotherapy with Nab-P containing regimens (Nab-P plus gemcitabine or cisplatin, Nab-P, capecitabine, gemcitabine [PAXG] schedules) allowed by Agenzia Italiana del Farmaco [AIFA] regulation and Associazione Italiana di Oncologia Medica [AIOM] guidelines for PC treatment^7^, (5) availability of data on nutritional counseling (NC), NS, PERT administration, laboratory parameters, and outcomes of interest (survival, body weight, maldigestion-related symptoms), (6) completion of at least 3 months of first-line treatment, and (7) adequate follow-up. The analysis was notified to the local Ethics Committee [Comitato Etico Provinciale Area 1—A.O.U. di Foggia, ASL FG, ASL BAT]. A retrospective analysis was performed on anonymized data, and informed consent was not required due to the retrospective type of the study. The study protocol conforms to the ethical guidelines of the 1975 Declaration of Helsinki, as reflected in a prior approval by the institution’s human research committee.

### NS and PERT Administration

All patients received nutritional and body composition evaluation at baseline through clinical examination, Malnutrition Universal Screening Tool (MUST) and Bioelectric Impedance Analysis (BIA). NS was proposed to patients identified as malnourished or at risk of malnutrition according to the following criteria: (1) a score ≥2 according to MUST and (2) a phase angle <4.8° (female) and <5° (male). The NS was tailored for each patient according to European Society for Clinical Nutrition and Metabolism (ESPEN) guidelines^11^ as follows: (1) dietary counseling/advice, (2) oral nutritional supplements (ONS), (3) enteral/parenteral nutrition. Dietary advices were oriented to encourage the intake of protein- and energy-rich foods and fluids that are well tolerated. The additional use of ONS was advised when an enriched diet was not effective in reaching nutritional goals. Finally, if ONS remained inadequate despite nutritional interventions, enteral/parenteral nutrition was administered. Patients who showed a trend in improved MUST score (ie, increased BMI or weight for any value) or improved phase angle (ie, increased value) at the first follow-up control, were defined as “adequate” support. Conversely, patients who failed to obtain an improvement were still considered as malnourished or at risk of malnutrition and were treated with enteral/parenteral nutrition. PERT was administered according to each patient’s characteristics and needs, using capsules containing 10 000 U.Ph.Eur of lipase. As the new enzyme formulation containing 35 000 U.Ph.Eur of lipase became available, in February 2022, for exocrine pancreatic insufficiency, patients on PERT were allowed to switch from 10 000 to 35 000 capsules. Patients were considered adherent to PERT only if they had already started treatment at the beginning of first-line chemotherapy or started concomitantly. After baseline evaluation, monthly remote and 3-month clinical follow-ups were scheduled. Only adherent patients receiving at least 3 months of NS and PERT, according to the abovementioned criteria and subsequent nutritional follow-up visits were included in the NS/PERT cohort.

### Study Endpoints

The primary endpoint of this study was to explore the impact of NS and PERT use in terms of OS in patients with mPC undergoing first-line chemotherapy. The OS was defined as the time from first-line chemotherapy initiation to the date of death for any cause or last follow-up visit. Secondary endpoints were represented by the relevance of NS and PERT on patients’ body composition, weight, and maldigestion-related symptoms. The body composition-related parameters considered for this purpose were the free-fat mass index (FFMI) and phase angle. FFMI was defined as the amount of muscle mass in relation to height and weight (FFMI = fat-free mass [kg] (measured with BIA)/(height [m])². Phase angle was calculated by BIA as the ratio between reactance and electric resistance. The exploratory evaluation also included the impact of NS and PERT on progression-free survival (PFS), overall response rate (ORR), disease control rate (DCR), ­treatment-related toxicity, and rate of second or further lines of treatment. PFS was defined as the time elapsed from first-line chemotherapy initiation and disease progression or death for any cause or last follow-up visit. ORR and DCR were defined as complete responses (CR) + partial responses (PR) and CR + PR + stable diseases (SD), respectively. Finally, adherence to PERT was also evaluated as the number of capsules planned per day/number of capsules assumed per day.

### Assessments

Data on the patient’s diagnosis, medical history, and treatment were extracted from clinical records. Demographics and disease parameters evaluated in the final analysis were age, gender, Karnofsky performance status (KPS), primary tumor location (pancreatic head/uncinate process vs. body/tail), BMI, weight, histology, grading, germline *BRCA* mutation, Tumor Node M﻿etastasis stage at diagnosis, previous treatment (neoadjuvant therapy, radical surgery, adjuvant therapy), and metastatic sites. Laboratory parameters considered for this study were baseline hemoglobin, lymphocyte count, neutrophil-to-lymphocytes ratio, transferrin, albumin, fasting serum glucose, cholesterol, and Ca19.9. All these parameters were evaluated at baseline (day 1 of first-line chemotherapy). Initial assessment also included nutritional items (FFMI and phase angle) and maldigestion-related symptoms through questionnaires. In particular, at each visit, patients were asked to indicate the presence or absence of symptoms that could be attributable to maldigestion (appetite loss, feeling of indigestion, bloating, frequent stools, floating or greasy/fatty in stool).

Treatment and outcomes evaluation included schedule, number of cycles, best response (according to RECIST 1.1 criteria), ORR, DCR, OS, PFS, and toxicity (according to Common Terminology Criteria for Adverse Events [CTCAE] version 5.0). KPS, weight, FFMI, phase angle, ­maldigestion-related symptoms, and Ca19.9 were also assessed at 3 months.

### Statistical Analysis

Data were summarized by descriptive analysis. Normality data distribution was tested by Kolmogorov-Smirnov test. Means, median, and standard deviations (SDs) were calculated for continuous variables, while absolute values and frequency (percentage) were calculated for categorical variables. Comparison of mean values between groups was performed by a Student’s *t* test, comparison of mean values within group was performed by the Paired *t* test and comparison of proportions by the Chi-square test. For investigating PFS and OS, the Kaplan-Meier curves and the log-rank test were used. To investigate the association between the survival time of patients and some predictor variables, the Cox ­proportional-hazard model was used. All analyses were performed with IBM SPSS Statistics for Windows version 28.0.1.1.

## Results

### Baseline Patient and Cancer Characteristics

Overall, 106 consecutive patients were included in the study, of whom 53 were in the NS/PERT cohort and 53 were not (with approximately 2/3 of males and 1/3 of females in both groups). The overall median age was 67 years (range 52-86). No significant differences were observed between the 2 cohorts in terms of KPS. Cancer features, including primary tumor site (head/uncinate process vs. body/tail), metastatic site, histologic type, grading, the incidence of *BRCA* mutations and disease stage at diagnosis, were not significantly different between the groups (Table 1).

**Table 1.** Demographic data and cancer characteristics at baseline.

	Without PERT/NS *N* = 53 (%)	With PERT/NS *N* = 53 (%)	Total *N* = 106 (%)	*P*-value
Female	16 (30.2%)	21 (39.6%)	37 (34.9%)	.208
Male	37 (69.8%)	32 (60.4%)	69 (65.1%)	
Age (years)	66.9 (± 8.8)	68.5 (± 8.5)	67.7 (± 8.7)	.326
Karnofsky PS	83 (± 8)	85 (± 9)	84 (± 9)	.366
*Primary tumor site*				
Body and tail	28 (52%)	24 (45.2%)	52 (49%)	.334
Head/uncinate	25 (48%)	29 (55%)	54 (51%)	
Process				
*Histology*				
PDAC	47 (89%)	47 (89%)	94 (89%)	.955
Other	6 (11%)	6 (11%)	12 (11%)	
*Grading*				
G2	6 (11.3%)	13 (24.6%)	19 (17.9%)	
G3	32 (60.4%)	29 (54.7%)	61 (57.5%)	.242
G4	3 (5.7%)	4 (7.5%)	7 (6.6%)	
NA	12 (22.6%)	7 (13.2%)	19 (17.9))	
BRCA (*N* = 65)	*N* = 12	*N* = 53		
*BRCA1* mutation	1 (8.3%)	4 (7.5%)	5 (7.7%)	.790
*BRCA2* mutation	0	2 (3.8%)	2 (3.1%)	
No mutation	11 (91.7%)	47 (88.7%)	58 (89.2%)	
*Stage at diagnosis*				
II	5 (9.4%)	6 (11.3%)	11 (10.4%)	.941
III	17 (32.1%)	16 (30.2%)	33 (31.1%)	
IV	31 (58.5%)	31 (58.5%)	62 (58.5%)	

*N* = 106.

NS = nutritional support; PERT = pancreatic enzyme replacement therapy; PS = performance status; PDAC = pancreatic ductal adenocarcinoma; NA = not applicable.

No differences were observed in metastatic sites between the 2 groups and, as expected, liver was the most involved metastatic site (Supplementary Table S1). The mean values of laboratory tests were not significantly different between the groups (Supplementary Table S2).

Groups were similar for previous treatments, such as neoadjuvant therapy (chemotherapy, chemoradiotherapy), surgery, and adjuvant chemotherapy (Supplementary Table S3).

The Nab-P + gemcitabine and PAXG regimens had similar frequency (49% vs. 51%) in the NS/PERT group of patients, while Nab-P + gemcitabine was more frequently used than PAXG (91% vs. 9%) in patients who did not receive NS/PERT (*P < .*001 between groups). Patients included in the NS/PERT group received a median of 6 (range: 1-12) cycles of chemotherapy, while those without NS/PERT received 4 (range: 1-22) cycles (*P = .*036) (Table 2).

**Table 2.** First-line therapies (*n* = 106).

	Without PERT/NS*N*=53 (%)	With PERT/NS *N* = 53 (%)	Total	*P*-value
Nab-P + G	48 (90.6%)	26 (49.1%)	74 (69.8%)	<.001
PAXG	5 (9.4%)	27 (50.9%)	32 (30.2%)	
Duration (months)	5.7 (±4.7)	6.5 (±2.9)	6.1 (±3.9)	.295
Median number of cycles (range)	4 (1-22)	6 (1-12)	6 (1-22)	.036

NS = nutritional support; G = gemcitabine; PERT = pancreatic enzyme replacement therapy.

### Nutritional and Clinical Outcomes

Among the 106 patients, all the 53 patients who received nutritional assessment, NC, and NS were treated with PERT. The mean adherence to PERT was 93 ± 7%. Among these patients, 28 switched from the 10 000 units formulation to the 35 000 U.Ph.Eur formulation. The mean adherence was higher after the switch (99.4 ± 2%, *P < .*001 at ­paired-samples *t* test).

Patients who received NS/PERT had proportionally higher weight gain at 3 months than patients who did not (*n* = 34, 64% vs. *n* = 20, 38%, *P = .*01). Accordingly, patients who received NS/PERT had a mean weight gain of 1.6 ± 3 kg, while those who received no NS/PERT had a negative mean weight change (−1.2 ± 2.9 kg) at 3 months, with a significant difference between groups (*P < .*001) (Supplementary Table S4).

Patients who received NS/PERT had a significantly higher mean phase angle at 3 months (5.5 ± 1.7) than at baseline (4.7 ± 1.5), *P < .*001. Consistently, the proportion of patients with a phase angle of <5° at 3 months was lower than at baseline (39% vs. 66%, *P < .*001) in this group. Similarly, the overall FFMI was significantly higher at 3 months than at baseline in the NS/PERT cohort (17.7 ± 1.8 vs. 16.9 ± 1.8, *P < .*001).

**Figure 1.**
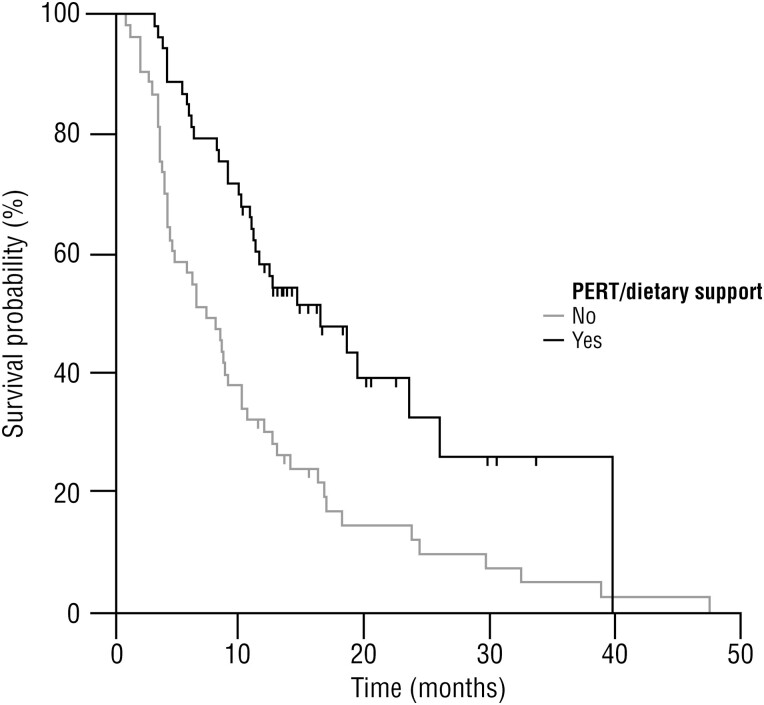
Kaplan-Meier curve of overall survival in patients with or without nutritional support (*n* = 106).

At the time of data censoring, 80 death events were recorded in the overall study population, of which 31/53 (58.5%) were in the NS/PERT group who received PERT and NS and 50/53 (94.3%) in the group without NS/PERT. The median OS was significantly higher in subjects who received NS/PERT than in the other group (16.5 [95% CI, 11.2 to 26.0] vs. 7.5 [95% CI, 4.5 to 10.3] months, *P < .*001; Fig. 1). The lack of NS/PERT was associated with a twofold increase in the risk of death (95% CI, 1.1-3.2, *P = .*013). In a binary logistic regression model a lower KPS at 3 months, the presence of multiple metastases and the absence of dietary supplementation are significantly related to the event “death” (Table 3).

**Table 3.** Correlation of mortality with clinical variables.

Variable	*P*-value	Odds ratio	95% CI
KPS	.007	0.9	0.8-1.0
Multiple metastases	.030	13.2	2.4-72.9
PAXG vs. Nab-P + G	.780	0.8	0.2-3.4
Without vs. with NS/PERT	.011	8.8	1.6-47.0
Constant	.007		

NS = nutritional support; G = gemcitabine; PERT = pancreatic enzyme replacement therapy; PAXG = gemcitabine or cisplatin, Nab-P, capecitabine, gemcitabine; KPS = Karnofsky performance status.

Overall, patients treated with PAXG had a significantly higher OS than subjects treated with Nab-P and Gemcitabine (14.7 vs. 8.7 months, *P = .*007); within the group of subjects treated with PAXG, the OS was significantly higher in subjects receiving NS/PERT (*P = .*004; Supplementary Fig. S1). Similarly, patients treated with Nab-P + Gemcitabine had longer OS, even if not statistically significant when receiving NS/PERT (*P = .*086; Supplementary Fig. S2).

At the time of data censoring, 99 patients in the overall study population had disease progression, of which 49/53 (92.5%) were in the group who received NS/PERT and 50/53 (94.3%) in the group who did not receive it. The median PFS was higher in the overall population of patients with NS/PERT than those without, although the difference was not significant (8 months vs. 4.9, respectively; *P = .*08; Supplementary Fig. S3).

Consistently, the ORR was higher, even if not significantly, in patients who received NS/PERT, being 41.5% and 24.5%, respectively (*P = .*098). Interestingly, DCR was 79% in patients with NS/PERT and 53% in patients without NS (*P = .*007; Table 4).

**Table 4.** Responses to first-line treatment (*N* = 106).

Best response	Without PERT/NS *N* = 53 (%)	With PERT/NS *N* = 53 (%)	Total	*P*-value
CR	2 (3.8%)	3 (5.7%)	5 (4.7%)	.037
PR	11 (20.8%)	19 (35.8%)	30 (28.3%)	.037
SD	15 (28.3%)	20 (37.7%)	35 (33.0%)	.037
PD	25 (47.2%)	11 (20.8%)	36 (34.0%)	.098
ORR	13 (24.5%)	22 (41.5%)	35 (33%)	.098
DCR	28 (52.8%)	42 (79.2%)	70 (66%)	.007

NS = nutritional support; PERT = pancreatic enzyme replacement therapy; CR = complete responses; PR = partial responses; SD = stable diseases; PD = progressive disease; ORR = overall response rate; DCR = disease control rate.

Decrease of Ca19.9 at 3 months was observed in 58.5% and 28.2% of patients in the NS/PERT and no-NS/PERT groups of patients, respectively (*P = .*008).

A higher proportion of patients received second-line chemotherapy in the group with NS/PERT than in the other (82% vs. 40%, *P < .*001).

No significant difference was observed in adverse events frequency either with or without NS/PERT, except for a higher number of hand-foot syndrome events among patients who received NS/PERT (*n* = 9, 17% with NS/PERT vs. *n* = 0 without NS/PERT; Supplementary Table S5).

### Impact of PERT and NS on Maldigestion-Related Symptoms and Performance Status

**Figure 2.**
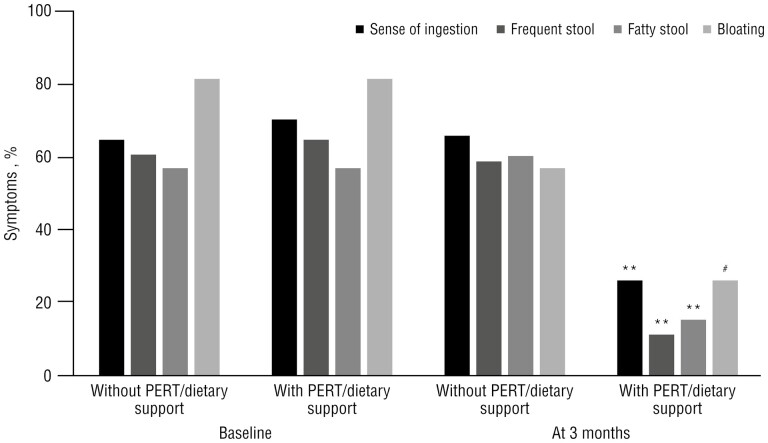
Digestive symptoms in patients who received and those who did not receive nutritional support (*n* = 106).**P < .001 vs. without PERT/nutritional support; #*P = .*003 vs. without PERT/nutritional support.

While there was no difference in the incidence of digestive symptoms in the 2 groups at baseline, the incidence of sense of indigestion, frequent stool, fatty stool, and bloating were significantly lower in patients with NS/PERT than in the other group (*P < .*001 for all except bloating, *P = .*003; Fig. 2). The KPS was similar at baseline in the 2 groups, but it was higher in the group with NS/PERT after 3 months (83 ± 11% vs. 70 ± 16%, *P < .*001). The mean KPS had no significant changes in patients receiving NS/PERT, while it decreased significantly after 3 months compared with baseline (83 ± 8% at baseline and 70 ± 16% at 3 months, *P < .*001) in those with no NS/PERT.

Among patients who reached disease control, the KPS had no significant change at 3 months compared with baseline; it was higher in patients with NS/PERT than in those without after 3 months (87 ± 7% vs. 80 ± 12%, *P = .*006). On the contrary, in patients without disease control (DC), there was an overall lower mean KPS at 3 months compared with baseline (71 ± 3.5% vs. 79 ± 8%, *P < .*001), with no significant difference between the 2 groups of patients (Table 5). So, the patients who obtained DC, after 3 months maintained a KPS similar to baseline score if receiving NS/PERT (baseline 86.4 ± 8.2 and 87 ± 7 at 3 months), while KPS was slightly reduced without a NS/PERT (80 ± 12). On the contrary, patients who did not reach DC had a reduced KPS at 3 months independently of the NS/PERT (76.6 ± 8 at baseline, 71 ± 3 at 3 months without diet, and 73 ± 5 at 3 months with NS, Table 5).

**Table 5.** Karnofsky PS analysis at baseline and after 3 months (*N* = 106).

Patients with NS/PERT	Baseline	3-months		*P*-value
Mean (SD)	84.5 (±9.1)	83.0 (±10.8)		.159
Patients without NS/PERT	Baseline	3 months		*P*-value
Mean (SD)	83.0 (±8.0)	70.0 (±15.8)		<.001
Patients with DC	Baseline (*N* =70)	3 months (*N* = 70)		*P*-value
Mean (SD)	86.4(±8.2)	85.0 (±8.5)		.142
Patients without DC	Baseline (*N* = 36)	3 months (*N* = 36)		*P*-value
Mean (SD)	78.6 (±8.0)	71.4 (±3.5)		<.001
KPS at 3 months, with DC	Without NS/PERT (*N* = 28)	With NS/PERT (*N*= 42)	Total (*N*=70)	*P*-value
Mean (SD)	80 (±12)	87 (±7)	85 (±8)	.006
KPS at 3 months, without DC	Without NS/PERT (*N* = 25)	With NS (*N* = 11)	Total (*N*=36)	*P*-value
Mean (SD)	71 (±3)	73 (±5)	71 (±4)	.131

NS = nutritional support; PERT = pancreatic enzyme replacement therapy; KPS = Karnofsky performance status; DC = disease control.

## Discussion

This retrospective study evaluated the impact of NS and PERT both on clinical outcomes and nutritional parameters in patients with mPC undergoing first-line chemotherapy with Nab-P containing schedules. In such a context, this study investigated the unmet need to integrate nutritional therapy as a crucial part of the multimodal care process in patients with PC, as recently recommended by a Spanish multidisciplinary panel.^12^ Our results showed that PERT and simultaneous dietary interventions are related to longer OS and have a significant, independent, prognostic impact for better outcomes. This beneficial effect of NS/PERT was independent from the therapeutic strategy. In addition, a higher proportion of patients obtained DC with NS/PERT than without it, and the median PFS was higher in patients receiving NS/PERT.

We did not observe differences in toxicity profile between patients who received PERT and NS and those who did not, except for a higher incidence of hand and foot syndrome. This event was significantly higher in the NS/PERT cohort, and this may be explained by the more frequent use of capecitabine contained in the PAXG schedule in this subgroup. Similarly, the trend (*P = .*053) to increased incidence of G3/4 fatigue in the NS/PERT group may be related to the higher number of patients treated with PAXG that is slightly more toxic than Nab-P plus gemcitabine.^6^ This observation may appear in contrast with the general idea that fatigue is often related to cancer-induced cachexia. Indeed, in our study, a major observation is that simultaneous use of PERT and NS prevented weight loss and KPS deterioration during chemotherapy and obtained an improvement of nutritional parameters, such as phase angle and FFMI, after 3 months of anticancer treatment. Therefore, we suggest that the higher incidence of fatigue in the NS/PERT cohort may be related to the higher toxicity of the 4-drug regimen.

Noteworthy, this pro-active approach, the early nutritional assessment with MUST and BIA, along with clinical evaluation and the subsequent “tailored” nutritional intervention according to the ESPEN guidelines, allowed to improve nutritional results. Our findings with the use of NS are aligned with recent reviews suggesting shorter length of stay in hospital, lower rate of complications, and lower weight loss with enteral vs. parenteral nutrition in patients with PC.^13,14^ Consistently, we observed a lower incidence of maldigestion- related symptoms in patients who were strictly adherent both to PERT and NS. This may facilitate oral intake of nutrients, that results in adequate nutrient intake, as suggested by the ESPEN guidelines.^11^ A recent meta-analysis of 4 randomized controlled trials with the use of PERT in advance patients with PC found no significant difference in OS (standardized mean difference [SMD] 0.12; 95% CI, −0.46 to 0.70, *P = .*046) as well as in change in body weight (SMD 0.53; 95% CI, −0.72 to 1.77; *P = .*21). However, this analysis was limited by non-uniform trial designs and different end points, along with small numbers of patients.^15^

Our results are consistent with those obtained by Trestini et al,^16^ in a retrospective study including 110 patients with advanced pancreatic ductal adenocarcinoma treated with first-line Nab-P + gemcitabine. These authors observed that previous surgical resection of the primary tumor (hazard ratio [HR]: 2.67, *P = .*11) and weight gain after 3 months (HR: 1.68, *P = .*07), and PERT (HR: 2.85, *P* ≥ .001) were independent predictors of OS. In addition, patients who received PERT improved maldigestion-related symptoms at 3 months more frequently than patients who did not (85.2% vs. 14.8%, *P* ≥ .0001).

As the new enzyme formulation containing 35 000 U.Ph.Eur of lipase became available, in February 2022, for exocrine pancreatic insufficiency, patients on PERT were allowed to switch from 10 000 to 35 000 capsules. Although we have no adequate data to draw conclusions, our study represents the first report about the use of this novel formulation and suggests that the introduction of the 35 000 units of lipase formulation may further improve the adherence to PERT, because of a lower number of capsules/day for patients.

Noteworthy, NS/PERT intervention prevented the deterioration of nutritional parameters and KPS, and this resulted in a parallel improvement of oncological outcomes, as response rate and survival. Interestingly, this is consistent with previously observations that reduced FFMI independently predicted survival and was associated with impaired quality of life.^17^ This appears to be particularly true in the subgroup of patients who obtained a disease control with anticancer treatments. In this subgroup, KPS was not globally changed after 3 months. However, it was higher in subjects who received NS/PERT and lower in patients who did not, suggesting that the overall maintenance of mean KPS at 3 months should be attributed to the positive impact of PERT and NS. On the contrary, KPS was significantly reduced after 3 months in non-responding patients, both in those who had received NS/PERT and those who had not. In the light of this observation, we may speculate that patients whit DC may truly benefit from NS/PERT in addition to anticancer therapy to maintain a good performance status. This conclusion is also supported by the observation that patients who received both PERT and NS received a higher number of cycles of therapy in first line and higher proportion of further lines of treatment. These results support the conclusion that NS and prevention of nutritional deterioration may represent a prerequisite to improve patients’ well-being and oncological outcomes. A recent systematic review investigating different nutritional approaches in patients with PC, confirmed the importance of such interventions (counseling, enteral, or parenteral nutrition) but was not conclusive due to the heterogeneous nature of the randomized controlled trials conducted so far in this area.^13^ Thus, the major strength of this study, although retrospective and with the limitation of a relative small number of patients, is represented by the concomitant evaluation of both NS and PERT in the same cohort. In fact, to our knowledge, this represents the first report describing the effects of both approaches in a group of patients affected by mPC undergoing first-line chemotherapy.

Finally, based on this experience, our data, along with the opinion of Rovesti et al,^18^ suggest that detection and management of PC-related malnutrition syndromes are of primary importance and deserve a specific and multidisciplinary (clinical nutrition, oncology, gastroenterology) approach to improve survival, but also the quality of life. Further prospective clinical trials evaluating the clinical nutrition, simultaneously with PERT in patients with mPC undergoing to first-line chemotherapy are largely awaited to better understand the real impact of this approach. In our opinion, randomized controlled trials, with an adequate sample size and statistical power to detect significant and clinically meaningful differences oriented both on survival, nutritional parameters, and quality of life should be designed.

## Conclusions

To our knowledge, these data, with the limitations of a retrospective study performed on a relative small cohort, represent the first report about the impact of both NS and PERT in patients with mPC undergoing first-line chemotherapy.

Although the aforementioned limitation, the results from this study reinforce the need for a multimodal and multidisciplinary approach from the beginning and suggest that supportive, simultaneous therapies may have a potential, significant clinical impact in mPC management, being able to preserve patients performance status, and increase their survival. In our population, patients who receive both PERT and NS from the beginning of treatment had a longer OS, higher disease control and better body composition parameters. In addition, PERT showed the potential to improve ­maldigestion-related symptoms, reduce weight loss, and prevent KPS deterioration. Therefore, more awareness about the use of PERT in this setting and further high-quality studies are needed.

## Supplementary Material

oyad101_suppl_Supplementary_Materials

## Data Availability

The data underlying this article are available in the article and in its online Supplementary material.
