# Supplementary material for: Pancreatic Enzyme Replacement and Nutritional Support With n﻿ab-Paclitaxel-based First-Line Chemotherapy Regimens in Metastatic Pancreatic Cancer
Source: Oncologist. 2023 May 8;28(9):e793–800. doi: 10.1093/oncolo/oyad101 (PMC10485404; doi:10.1093/oncolo/oyad101)
Supplement: oyad101_suppl_Supplementary_Materials [file oyad101_suppl_supplementary_materials.zip › Supplementary Tables 1-5 final version GG-MLAS_230215.docx]

Supplementary Table 1. Sites of metastasis.

| **Site** | **Without PERT/nutritional support (n=53), n (%)** | **With PERT/nutritional support (n=53), n (%)** | **Total (n=106), n (%)** | **p-value** |
| --- | --- | --- | --- | --- |
| Liver | 37 (69.8%) | 33 (62.3%) | 70 (66.0%) | 0.539 |
| Bones | 3 (5.7%) | 1 (1.9%) | 4 (3.8%) | 0.618 |
| Lung | 21 (39.6%) | 12 (22.6%) | 33 (31.1%) | 0.093 |
| Peritoneum | 17 (32.1%) | 18 (34.0%) | 35 (33.0%) | 1.000 |
| Lymph nodes | 13 (24.5%) | 11 (20.8%) | 24 (22.6%) | 0.817 |

Supplementary Table 2. Laboratory tests.

| **Laboratory tests** | **Without PERT/nutritional support (n=53), mean ± SD** | **With PERT/nutritional support (n=53), mean ± SD** | **Total (n=106), mean ± SD** | **p-value** |
| --- | --- | --- | --- | --- |
| Hemoglobin | 11.8 ± 2.1 | 12.4 ± 2.4 | 12.1 ± 2.2 | 0.157 |
| NLR | 4.6 ±3.1 | 4.6 ± 2.2 | 4.6 ± 2.7 | 0.889 |
| LYMPH | 1.7 ± 0.7 | 1.8 ± 0.6 | 1.7 ± 0.7 | 0.498 |
| Transferrin | 327 ± 66 | 328 ± 65 | 328 ± 65 | 0.953 |
| Albumin | 3.3 ± 0.6 | 3.5 ± 0.6 | 3.4 ± 0.6 | 0.150 |
| Cholesterol | 157 ± 51 | 159 ± 51 | 158 ± 51 | 0.904 |
| Glucose | 121 ± 41 | 132 ± 40 | 126 ± 41 | 0.189 |

Supplementary Table 3. Previous therapies.

| **Previous therapies** | **Without PERT/nutritional support (n=53), n (%)** | **With PERT/nutritional support (n=53), n (%)** | **Total (n=106), n (%)** | **p-value** |
| --- | --- | --- | --- | --- |
| Neoadjuvant | 2 (3.8%) | 5 (9.4%) | 7 (6.6%) | 0.437 |
| Surgery | 11 (20.8%) | 17 (32.1%) | 28 (26.4%) | 0.271 |
| Adjuvant | 7 (13.2%) | 12 (22.6%) | 19 (17.9%) | 0.311 |
| Chemotherapy | 5 (9.5%) | 3 (5.7%) | 8 (7.5%) | - |
| Chemo-radiotherapy | 1 (1.9%) | 7 (13.2%) | 8 (7.5%) | 0.132 |
| Radiotherapy | 1 (1.9%) | 2 (3.8%) | 3 (2.8%) | - |

Supplementary Table 4. Body weight changes.

| **Weight** | **Without PERT/nutritional support (n=53)** | **With PERT/nutritional support (n=53)** | **Total (n=106)** | **p-value** |
| --- | --- | --- | --- | --- |
| Gain, n (%) | 20 (37.7%) | 34 (64.2%) | 54 (50.9%) | 0.011 |
| Mean variation (SD) | -1.2 ± 2.9 | 1.6 ± 3.0 | 0.2 ± 3.3 | <0.001 |

Supplementary Table 5. Adverse events.

| **Adverse events** | **Without PERT/nutritional support (n=48), n (%)** | **With PERT/nutritional support (n=49), n (%)** | **Total (n=97), n (%)** | **p-value** |
| --- | --- | --- | --- | --- |
| Hematological | 45 (93.8) | 46 (93.9) | 91 (93.8) | 1.000 |
| Gastrointestinal | 28 (58.3) | 20 (40.8) | 48 (49.5) | 0.106 |
| Other | 38 (79.2) | 47 (95.9) | 85 (87.6) | 0.015 |
| G3–4 hematological | 23 (47.9) | 29 (59.2) | 52 (53.6) | 0.312 |
| G3–4 gastrointestinal | 4 (8.3) | 4 (8.2) | 8 (8.2) | 1.000 |
| G3–4 other | 11 (22.9) | 14 (28.4) | 25 (25.8) | 0.644 |
| **Other adverse events** | **n=53** | **n=53** | **n=106** |  |
| Fatigue | 23 (43.4%) | 33 (62.3%) | 57 (53.8%) | 0.053 |
| Hand–foot syndrome | 0 | 9 (17.0%) | 9 (8.5%) | 0.002 |
| Increased liver enzymes | 4 (7.5%) | 4 (7.5%) | 8 (7.5%) | 1.000 |
| Mucositis | 12 (22.6%) | 15 (28.3%) | 27 (50.9%) | 0.065 |
| Neuropathy | 16 (30.2%) | 19 (35.8%) | 35 (33.9%) | 0.538 |
