# Supplementary material for: Pancreatic Enzyme Replacement and Nutritional Support With n﻿ab-Paclitaxel-based First-Line Chemotherapy Regimens in Metastatic Pancreatic Cancer
Source: Oncologist. 2023 May 8;28(9):e793–800. doi: 10.1093/oncolo/oyad101 (PMC10485404; doi:10.1093/oncolo/oyad101)
Supplement: oyad101_suppl_Supplementary_Materials [file oyad101_suppl_supplementary_materials.zip › Supplementary Figure Legends.docx]

**Supplementary Figure Legends**

**Supplementary Figure 1.** Kaplan-Meier curve of overall survival in patients treated with PAXG, either with or without a nutritional support (*n* = 32).

**Supplementary Figure 2.** Kaplan-Meier curves for overall survival in treated with Nab-P + gemcitabine (*n* = 74).

**Supplementary Figure 3.** Progression-free survival in patients with and without nutritional support (*n* = 106).
