# Supplementary figures and images for: Pancreatic Enzyme Replacement and Nutritional Support With n﻿ab-Paclitaxel-based First-Line Chemotherapy Regimens in Metastatic Pancreatic Cancer
Source: Oncologist. 2023 May 8;28(9):e793–800. doi: 10.1093/oncolo/oyad101 (PMC10485404; doi:10.1093/oncolo/oyad101)

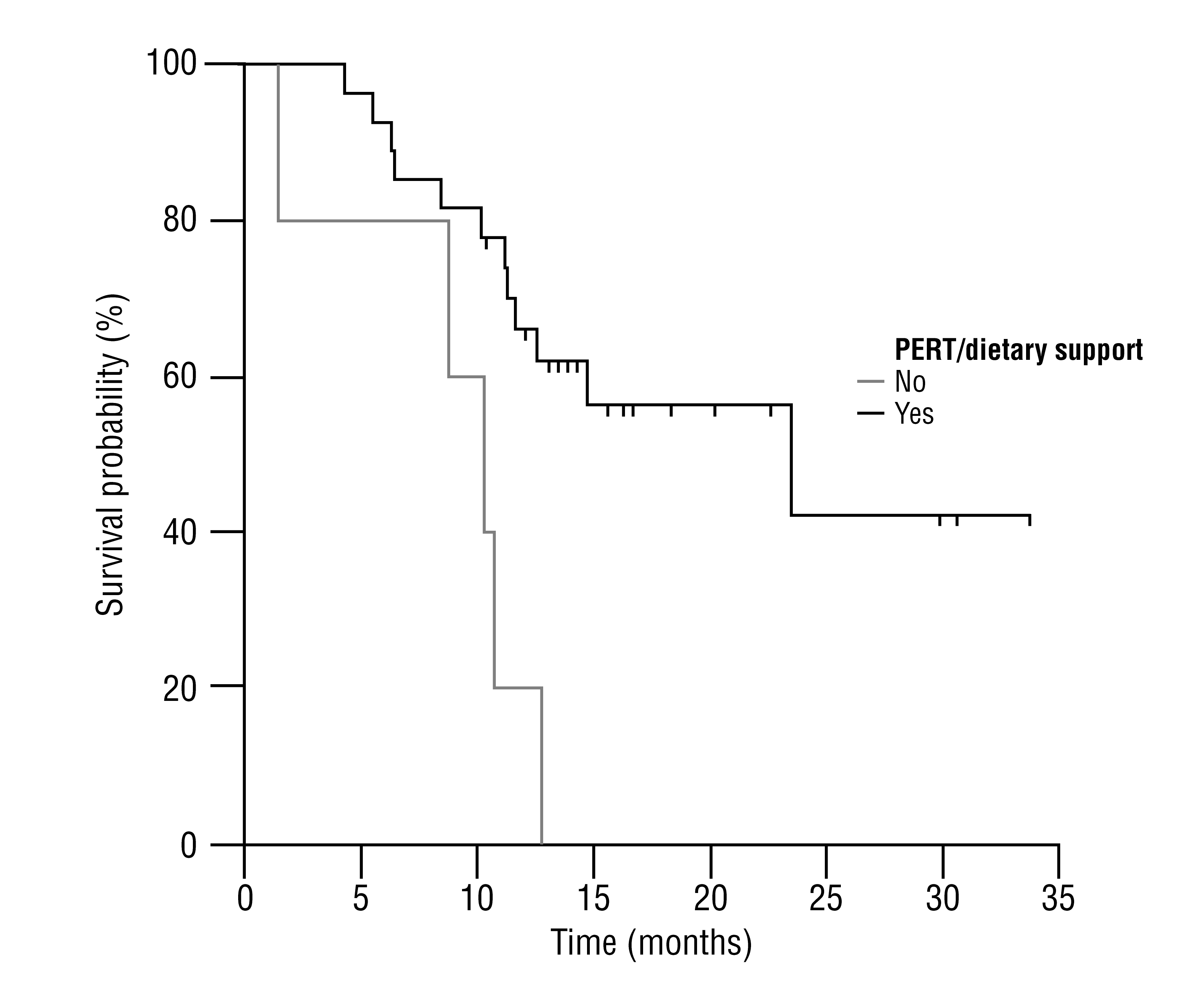

Supplement: oyad101_suppl_Supplementary_Materials [file oyad101_suppl_supplementary_materials.zip › Supplementary Figure 1.tif]

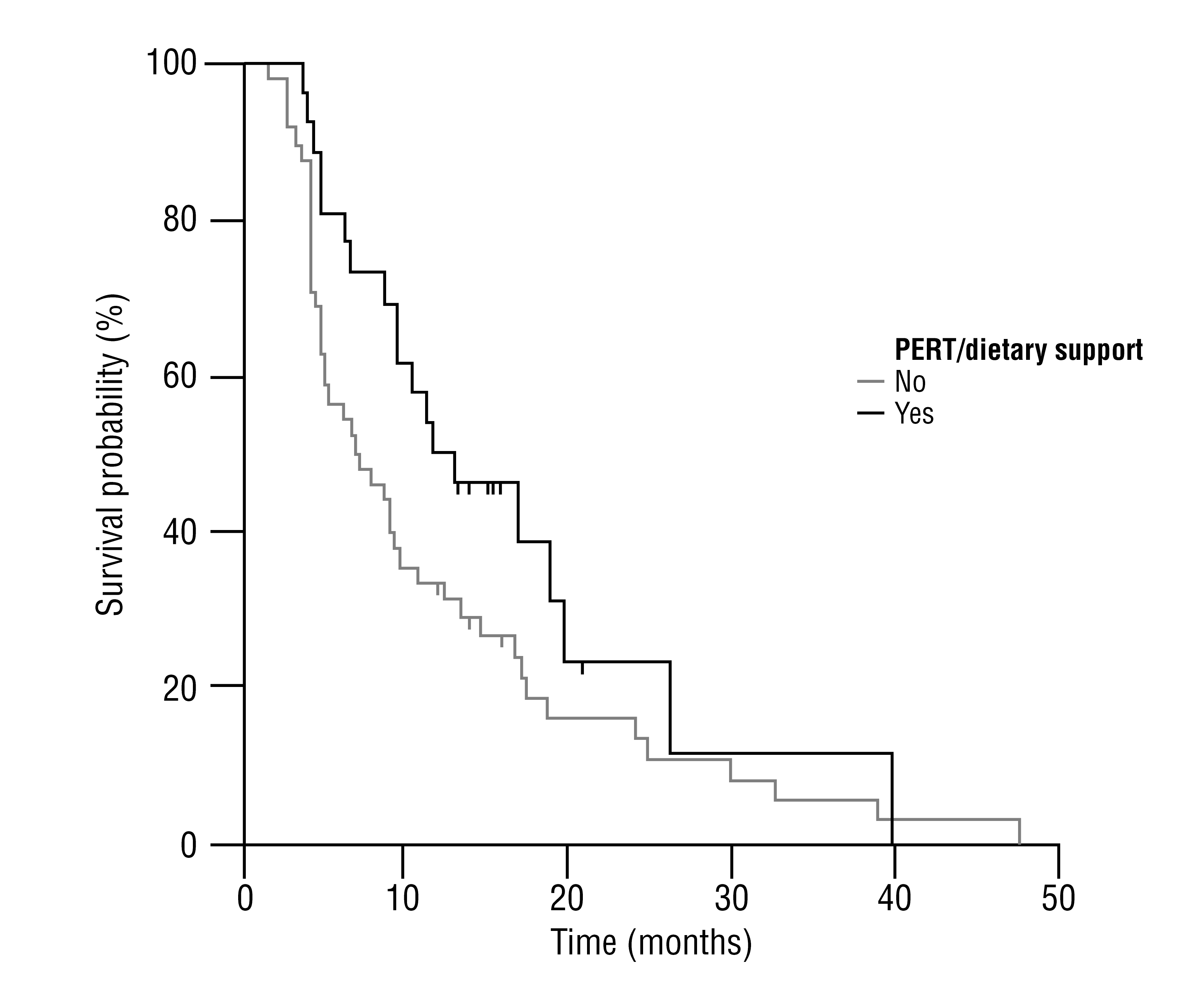

Supplement: oyad101_suppl_Supplementary_Materials [file oyad101_suppl_supplementary_materials.zip › Supplementary Figure 2.tif]

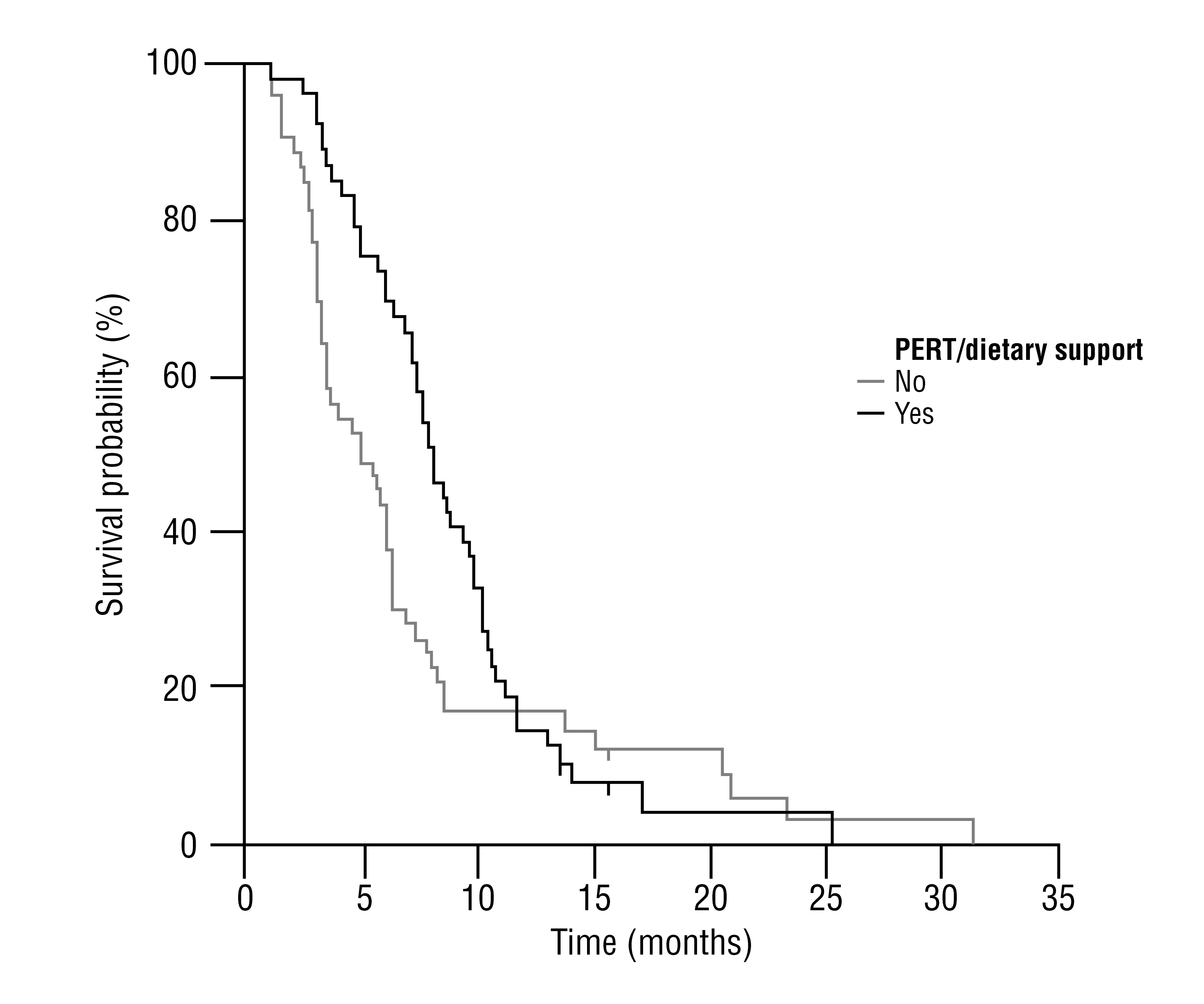

Supplement: oyad101_suppl_Supplementary_Materials [file oyad101_suppl_supplementary_materials.zip › Supplementary Figure 3.tif]
